# X-Ray Structure and Mutagenesis Studies of the *N*-Isopropylammelide Isopropylaminohydrolase, AtzC

**DOI:** 10.1371/journal.pone.0137700

**Published:** 2015-09-21

**Authors:** Sahil Balotra, Andrew C. Warden, Janet Newman, Lyndall J. Briggs, Colin Scott, Thomas S. Peat

**Affiliations:** 1 CSIRO Land and Water Flagship, Black Mountain, Canberra, Australia; 2 Research School of Chemistry, Australian National University, Canberra, Australian Capital Territory, Australia; 3 CSIRO Biomedical Manufacturing, Parkville, Australia; Monash University, AUSTRALIA

## Abstract

The *N*-isopropylammelide isopropylaminohydrolase from *Pseudomonas* sp. strain ADP, AtzC, provides the third hydrolytic step in the mineralization of *s-*triazine herbicides, such as atrazine. We obtained the X-ray crystal structure of AtzC at 1.84 Å with a weak inhibitor bound in the active site and then used a combination of *in silico* docking and site-directed mutagenesis to understand the interactions between AtzC and its substrate, isopropylammelide. The substitution of an active site histidine residue (His249) for an alanine abolished the enzyme’s catalytic activity. We propose a plausible catalytic mechanism, consistent with the biochemical and crystallographic data obtained that is similar to that found in carbonic anhydrase and other members of subtype III of the amidohydrolase family

## Introduction

Since its introduction in the 1950s, atrazine has been used extensively for weed control in corn, sorghum and sugarcane, which has resulted in the detection of atrazine in ground and surface water at concentrations of up 4.6 μM. At such concentrations, atrazine is reportedly responsible for endocrine disruption in vertebrates, causing chemical feminization in frogs [1]. The availability of high concentrations of atrazine in the environment has promoted the evolution of new metabolic pathways in bacteria that allow the use of atrazine as a carbon and nitrogen source [2–5]. Amongst the atrazine mineralization pathways that have evolved, the one from *Pseudomonas* sp. strain ADP has been studied most extensively [6–8]. This pathway mineralizes atrazine *via* the step-wise removal of the side chains (chlorine, ethylamine and isopropylamine) by the sequential action of three hydrolases (AtzA, AtzB and AtzC, respectively) followed by the hydrolytic decomposition of the ring by a further three hydrolases (AtzD, AtzE and AtzF) [9,10]. Genetic and biochemical studies have been conducted with each of these enzymes [11–20], and more recently there has been considerable interest in obtaining their X-ray crystal structures, with the structures of AtzA [21], AtzD [9], AtzF [22] reported. Additionally the structure of a functional analogue of AtzA, termed TrzN [23,24], has also been reported.

**Fig 1:**
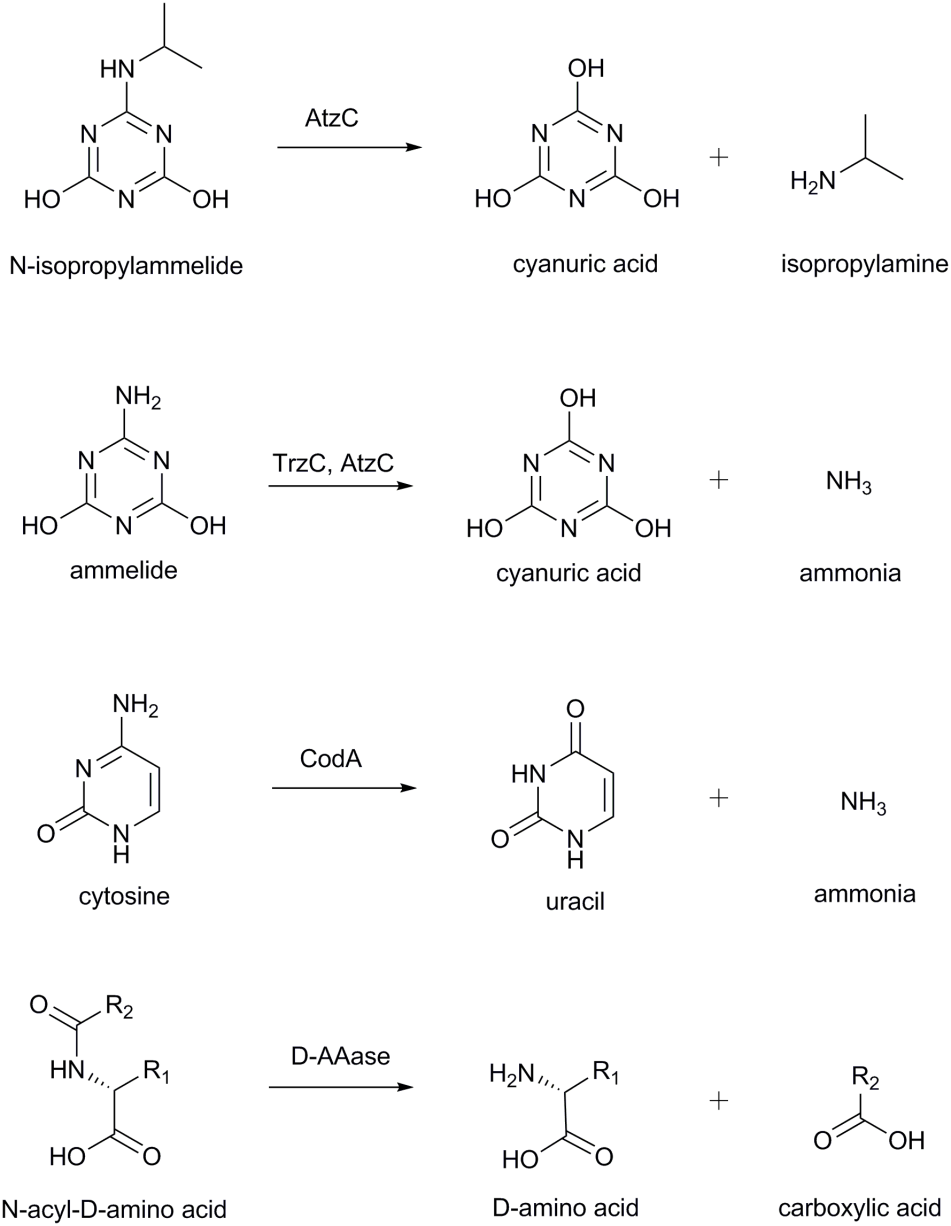
Reactions catalyzed by AtzC and its closest relatives, TrzC, CodA and D-AAase.

A structure for AtzC has also been deposited in the PDB (2QT3)[25]; AtzC is an isopropylammelide isopropylaminohydrolase (E.C. 3.5.99.4), catalyzing the hydrolysis of *N*-isopropylammelide (IPA) to form isopropylamine and cyanuric acid in the third step of atrazine catabolism (Fig 1) [15]. AtzC is a member of amidohydrolase protein superfamily and is the third enzyme of the atrazine mineralization pathway. It had been demonstrated by biochemical analysis that AtzC contains one Zn^2+^ ion per monomer, which is also seen in 2QT3 [15,25].

AtzC has 30–35% amino acid sequence identity with three enzymes of known function, ammelide aminohydrolase (TrzC, 3.5.3.-; 31% identity), bacterial cytosine deaminase (CodA, E.C. 3.5.4.1; 35% identity), and N-acyl-D-amino acid deacylase (D-AAase, 3.5.1.81; 35% identity). TrzC is involved in hydrolysis of the amine substituent of ammelide (2-amino-1,3,5-triazine-4,6-dione, Fig 1) in the melamine (1,3,5-triazine-2,4,6-triamine) mineralization pathway [26]. CodA is involved in the pyrimidine salvage pathway and deaminates cytosine to produce uracil and ammonia (Fig 1) [27]. D-AAases are involved in hydrolysis of N-acyl-D-amino acids to their corresponding D-amino acids and acetate (Fig 1) [28]. The structures and reaction mechanisms of CodA and D-AAase are well characterized [28,29]. The X-ray structure of AtzC deposited by Fedorov *et al*. [25] was obtained at moderate resolution (2.24 Å) without bound substrate or inhibitor. In this report, we present a 1.84 Å structure of AtzC with an inhibitor in the active site; this has guided substrate docking and mutagenesis experiments that have allowed us to propose a plausible reaction mechanism.

## Methods and Materials

### DNA manipulation

The unmodified *atzC* gene from *Pseudomonas* sp. strain ADP (accession number: U66917.2)[10] was obtained from GenScript (New Jersey, USA) as a pUC57 derivative. The *atzC* gene was then subcloned into pETCC2, an expression vector derived from pET14b that is described elsewhere [9] using *Nde*I and *Bam*HI restriction sites that had been included in the synthetic gene, but which did not affect the coding sequence. Mutagenesis was carried out using the method of overlap PCR of Ho *et al*. [30]. The list of primers used in this study is given in Table A in S1 File. The reaction conditions for PCR were 1× Phusion HF Buffer, 2 picograms pETCC2-*atzC* DNA template, 0.5 μM primers, 200 μM dNTPs and a half unit of Phusion DNA polymerase in a total reaction volume of 25 μL. The cycle conditions for the PCR reaction were a 30 s denaturation step at 98°C followed by annealing at 53°C for 20 s and then extension for 120 s at 72°C for 30 cycles.

The size confirmation and separation of the resulting amplicon was done on 0.6% agarose gel by electrophoresis. The DNA band was excised from the gel and purified using a NucleoSpin Gel and a PCR Clean-up kit (Macherey-Nagel). The amplicons were digested with *Bam*HI and *Nde*I restriction enzymes as per manufacturer’s instructions and purified by gel extraction as described above. The digested amplicon was cloned into the *Bam*HI and *Nde*I restriction sites of pETCC2 using T4 DNA ligase as per the manufacturer’s instructions. The DNA polymerase, dNTPs, T4 ligase and restriction enzymes were purchased from New England Biolabs (Ipswich, USA).

### Protein expression and purification

The *atzC* gene or its modified derivatives in pETCC2 were used to transform electrocompetent *Escherichia coli* BL21 (λDE3) cells (Invitrogen). The cells were grown at 310 K on Luria–Bertani (LB) agar plates [31] supplemented with 100 μg.mL^−1^ ampicillin. A single colony was picked from these plates and was used to inoculate a 50 mL starter culture using LB[31] as growth media supplemented with 100 μg.mL^−1^ ampicillin. This starter culture was kept at 310 K overnight whilst shaking at 200 rev.min^−1^ and the next morning was subsequently diluted to 1:20 into LB, Miller medium [31] supplemented with 100 μg.mL^−1^ ampicillin. The diluted culture was shaken at 310 K and 200 rev.min^−1^ and upon reaching an OD_600_ of 0.6–0.8, it was induced for protein expression by the adding isopropyl β-d-1-thiogalactopyranoside (IPTG) at 100 μM final concentration. The induced culture was kept at 310 K overnight whilst shaking at 200 rev.min^−1^.

The cells were harvested from the overnight culture by centrifugation at 4000 *g* for 10 min in an Avanti J-E centrifuge (Beckman Coulter, Indianapolis, USA) and then resuspended in 15 mL lysis buffer (50 mM TRIS pH 7.5, 100 mM NaCl) per liter of harvested culture. The resulting cell suspension was lysed by passage through an Avestin C3 homogenizer three times at 124 MPa. After cell lysis, the insoluble cellular debris was removed by centrifugation at 21,000 *g* using an Avanti J-E centrifuge. Wild-type AtzC was purified from the soluble cell-free extract in two steps: His-tag affinity chromatography using an Ni–NTA Superflow cartridge (Qiagen, Maryland, USA) with a gradient of 0–500 mM imidazole in 50 mM TRIS (pH 7.5) and 100 mM NaCl, and size-exclusion chromatography using a 130 mL column packed with Superdex 200 prep-grade resin (GE Healthcare Life Sciences) with a buffer comprised of 50 mM TRIS (pH 7.5), 100 mM NaCl. Twenty column volumes of buffer were used to achieve the gradients used for the Ni–NTA Superflow cartridge. The protein was concentrated in an Amicon Ultra-15 Centrifugal Filter Unit with an Ultracel-30 membrane (Millipore, Carrigtwohill, Ireland) to 10 mg.mL^−1^ and snap-frozen in liquid nitrogen in 100 μL aliquots. The final purity was estimated to be 98% from a Coomassie-stained gel. The mutants were purified by His-tag affinity chromatography using a 1 ml Ni–NTA Superflow cartridge (Qiagen) with a five column wash by 25 mM imidazole in 50 mM HEPES (pH 7.5) followed by eight column volume of second wash with 50 mM imidazole in the same buffer and finally eluted with six column volume of 250 mM imidazole in the same buffer.

### X-ray crystallography and data collection

2QT3 was described as “complexed with zinc”, and crystallized out of 2-methyl-2,4-pentanediol at pH 5.5 at room temperature (Screen JCSG+ condition H5). Based on this, the purified AtzC protein was thawed, supplemented with 1 mM ZnCl_2_, and set up against JCSG+ screen at two temperatures. This gave some indications of crystallization, at 281 K but not at 293 K, and suggested that the protein was over-concentrated. Subsequent trials were set up with the JCSG+ screen diluted to half strength with water, as well as the PACT screen (also at half strength), with zinc-supplemented protein at 10 mg.mL^-1^. Initial crystallization hits were all from trials containing polyethylene glycols. An optimization screen using the Optisalts additive screen (Qiagen) and a base condition of 0.1 M lithium sulfate, 0.05 M phosphate-citrate pH 4.2, 10% w/v PEG 1000 gave a crystal that resulted in a sub-3 Å diffraction data set.

Crystals of the tagged protein were generally poorly ordered, and subsequent trials focused on protein treated *in situ* with thrombin, to remove the N-terminal His-tag.

100 μL of tagged AtzC protein at 10 mg.mL^-1^ in 50 mM HEPES pH 7.5, 100 mM NaCl was added to a PCR tube containing 10 μg of lyophilized thrombin, and subsequently set up (without further purification) in JCSG+ and PACT screens. Well diffracting crystals were obtained from condition F9 of JCSG+ (2.4 M sodium malonate pH 7; Figure A in S1 File). Malonate grown crystals of thrombin treated protein (using protein at either 5 or 10 mg.mL^-1^) were used for soaking experiments, where atrazine, ammeline, barbituric acid, 2-hydroxyatrazine and ammelide were dissolved in either water or DMSO and added to the formed crystals. In all cases, the subsequent X-ray diffraction analysis showed malonate in the active site.

Crystals of the mutants His219Ala, His249Ala, Asp188Ala, Asn304Asp and Trp309Phe were grown in the same way as the native protein (*in-situ* thrombin treatment of the protein; using sodium malonate as the crystallant).

Malonate grown wild-type and mutant crystals were cryo-cooled (without the addition of further cryo-protectant) by plunging into liquid nitrogen, taken to the Australian Synchrotron MX-2 beamline and placed in the N_2_ stream at 100 K for data collection. 360 one degree images were taken from each crystal to obtain a complete data set. The reflections were indexed using XDS [32] and scaled using Aimless [33]. 2QT3 was used as the model for molecular replacement with the program Phaser [34]. The model was subsequently rebuilt manually using Coot [35] and refined using Refmac [36]. For the wild-type protein crystals the data were 99.3% complete to a resolution of 1.84 Å and the final model had an R_work_ of 19.1% with an R_free_ of 22.9% (see Table 1 for the crystallographic statistics). The mutant structures were obtained using the native structure as the starting model, but otherwise the data were treated the same as the native.

**Table 1:** Crystallographic Data.

PDB	4CQB (WT)	4CQC (H219A)	4CQD (H249A)	5AKQ (N304D)
Space group	C2	C2	C2	C2
Cell dimensions				
*a*, *b*, *c* (Å)	106.5, 86.7, 114.2	106.2, 87.3, 114.4	106.3, 87.1, 113.9	106.2, 86.6, 114.1
α, β, γ (°)	90.0, 104.7, 90.0	90.0, 103.9, 90.0	90.0, 104.6, 90.0	90.0, 104.4, 90.0
Resolution (Å)	46.3–1.84 (1.94–1.84)	46.3–2.20 (2.32–2.20)	46.3–2.25 (2.37–2.25)	46.2–2.60 (2.72–2.60)
*R*_merge_	0.136 (0.703)	0.136 (0.799)	0.189 (0.954)	0.256 (1.044)
*R*_pim_	0.054 (0.285)	0.052 (0.306)	0.078 (0.391)	0.155 (0.632)
*CC1/2*	0.996 (0.820)	0.997 (0.852)	0.991 (0.699)	0.986 (0.663)
*I* / σ*I*	10.7 (2.7)	12.5 (2.8)	9.0 (3.0)	6.9 (2.1)
Completeness (%)	99.3 (95.1)	99.4 (98.9)	99.3 (98.6)	100 (100)
Redundancy	7.4 (6.8)	7.7 (7.7)	6.7 (6.8)	7.3 (7.4)
**Refinement**				
Resolution (Å)	40.4–1.84	46.3–2.20	46.3–2.25	46.2–2.60
Unique reflections	81,509	48,598	45,047	29,424
*R*_work_ / *R*_free_ (%)	19.1 / 22.9	19.6 / 22.3	16.6 / 19.4	20.5 / 24.1
No. atoms	6,923	6,544	6,717	6,314
Protein	6,392	6,362	6,448	6,276
Zn ions / other	2 and 39	2 and 32	2 and 40	2 and 2
Water	490	186	227	34
*B*-factors (Å^2^)	19.4	32.2	27.2	36.0
Protein	19.0	32.1	27.1	36.2
Zn ions / other	16.8 / 24.8	46.9 / 49.2	26.1 / 42.7	33.1 / 40.0
Water	24.3	28.6	25.8	21.0
R.m.s. deviations				
Bond lengths (Å)	0.019	0.010	0.008	0.019
Bond angles (°)	1.777	1.269	1.180	1.772

### Differential scanning Fluorimetry (DSF)

Thermal melt analyses, using a bank of formulations that varied pH, buffer chemical and salt concentration (“Buffer screen 9”) [37] were performed on the (tagged) wild-type, His219Ala and His249Ala proteins and analyzed using the program Meltdown. All three showed clean unfolding curves, and were quite stable (with T_m_ values of 333.75 K (+/- 0.08), 336.45 K (+/- 0.75) and 330.15 K (+/- 1.6) respectively) in the 50 mM HEPES pH 7.5, 100 mM NaCl standard formulation, but displayed quite different stability profiles across the different formulations tested (Figure B in S1 File).

### Enzymatic activity and pH dependence

AtzC activity was measured at 301 K using ammelide as a substrate. A 100 mM stock solution of ammelide (Sigma Aldrich, MO) was prepared in 100 mM sodium hydroxide and was subsequently diluted to the required concentrations using 200 mM sodium phosphate buffer at pH 7.5 supplemented with 10% v/v glycerol. The reaction was started by adding AtzC to a final concentration of 40.3 nM and the decrease in absorbance at 230 nM was measured using SpectraMax M2 spectrophotometer (Molecular Devices, California, USA). In experiments performed to determine the pH-dependence of AtzC, the ammelide concentration was 400 μM and a glutamate dehydrogenase (GDH)-coupled assay [38] was used to measure the rate of ammonia release. In these experiments, a pH range from 6.5 to 8.5 was tested. The V_max_ and *K*_M_ values were obtained by linear regression in GraphPad version 6.01 for windows (GraphPad Software, La Jolla California USA, www.graphpad.com; Figure C in S1 File).

Malonic acid is analogous to a fragment of AtzC’s native substrate (Figure D in S1 File). For testing malonic acid as an inhibitor (Figure E in S1 File), a stock solution was prepared of malonic acid in 200 mM phosphate buffer after which the pH was adjusted to 7.5. Dilutions of this malonic acid solution were made in 200 mM phosphate buffer at pH 7.5 prior to testing. For the assay, enzyme was added to the malonate dilutions followed by the addition of the ammelide substrate.

### Docking of substrate in active site

Isopropylammelide was manually positioned in the active site of AtzC, guided by the position of malonate found in the malonate-grown AtzC crystals (PDB 4CQB). Geometry optimization using density functional theory (DFT) was then performed on the active site to refine the positions of the substrate, residue side chains, the coordinated water and the Zn^2+^ center. Prior to optimization, His219 was epsilon protonated as it was within hydrogen bonding distance of the aromatic nitrogen atom on the substrate. His249 was delta protonated as the epsilon nitrogen was within hydrogen bonding distance of the metal-coordinated water. Positional constraints were placed upon backbone atoms of all residues and the side chains of Lys65, Asp127 and Asp303 were set to their charged states. The carbonyl groups of the backbone atoms were modelled as simple aldehydes. Calculations were performed using Dmol3 as implemented in Accelrys Materials Studio 7.0. The PBE functional [39] was employed in conjunction with the DND basis set (comparable to the 6-31G* basis set) and basis file 4.4. Defaults for a coarse-grained geometry optimization applied for all other parameters. To validate the method, docking was performed with malonate yielding a docking conformation near identical to that obtained in the crystal structure (Figure F in S1 File).

## Results and Discussion

### The structure of AtzC

**Fig 2:**
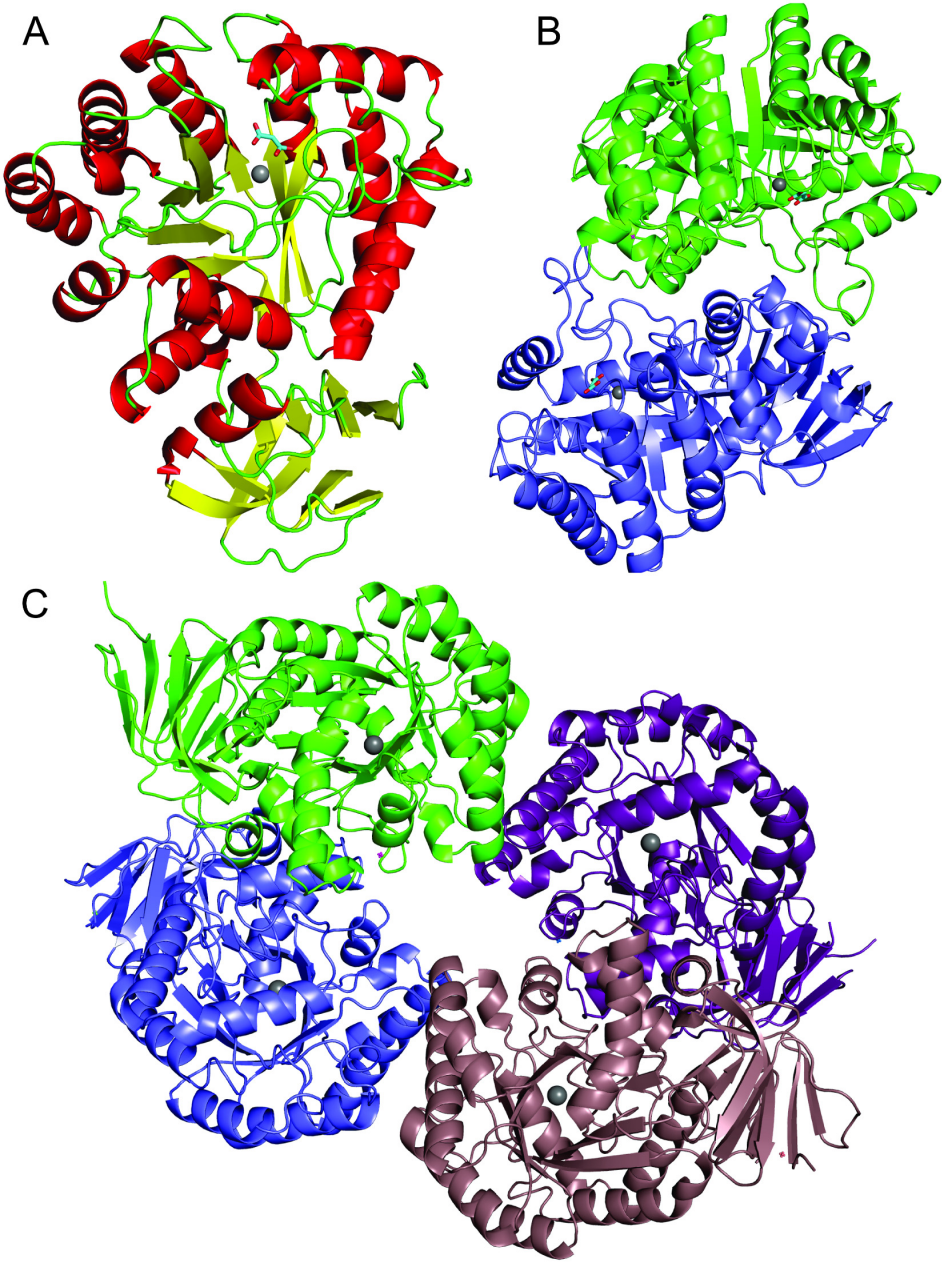
Structure of AtzC. A: A monomer of AtzC with the secondary structure highlighted by color: helices in red, strands in yellow and loops in green, with the Zn ion shown as a grey sphere. B: A dimer of AtzC and C: a tetramer of AtzC showing these interfaces with each monomer in a different color.

**Fig 3:**
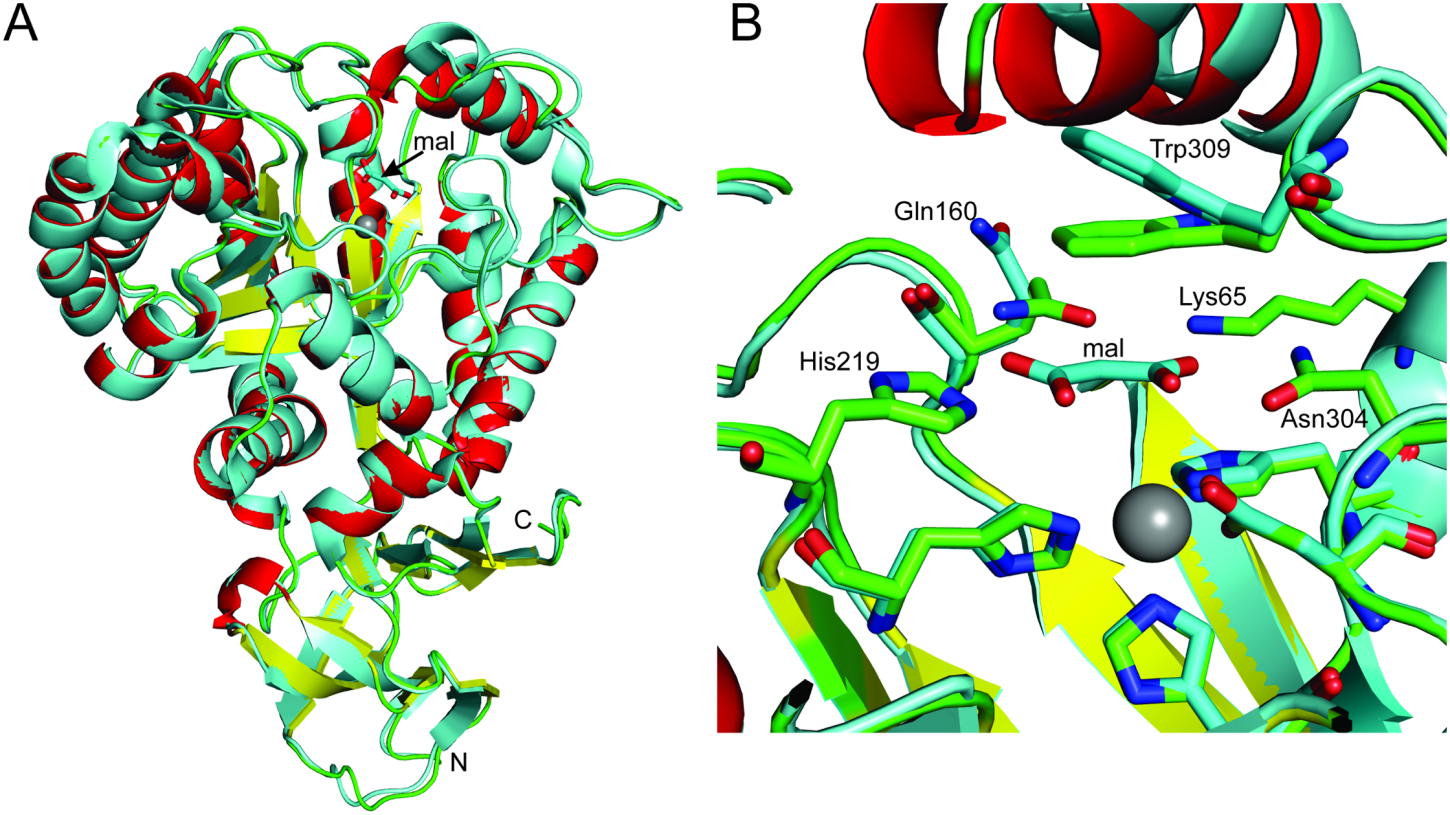
A comparison between the previously reported AtzC structure and the high resolution AtzC structure with bound inhibitor. A: The AtzC is shown as multicolored: helices in red, strands in yellow and loops in green whereas the 2QT3 structure is shown in cyan. The amino (N) and carboxy (C) termini of the proteins are indicated, as is the location of malonate (mal). B: Residues in the active site of AtzC are shown in stick representation and named along with malonate (mal). 2QT3 carbons are shown in cyan whereas the structure reported here have green carbons. The Zn ion is shown as a grey sphere coordinated to His60, His62, His217 and Asp303. The Trp309 and Gln160 residues are seen to be in a different orientation between the ligand bound and apo structures.

AtzC is a tetramer, according to PISA [40], our SEC data (native molecular mass of ~210 kDa), and previous reports [16], with the asymmetric unit containing half of the tetrameric structure (Fig 2). Each AtzC tetramer is a dimer of dimers (Fig 3C). The interface between monomers that form dimers occludes about 1870 Å^2^ of surface, whereas each of the interfaces between the two dimers of the tetramer are only 545 Å^2^. Each protomer is bilobal (Fig 3B), with the N- and C-termini coming together to form a small β-barrel (residues 2 to 55, then 358 to 403) with four β-strands each and a crossover strand (51–55) from the N-terminal side hydrogen bonding with the C-terminal sheet. The second and larger domain which contains the active site is a TIM-barrel fold (Fig 3A).

The malonate (a poor inhibitor of AtzC, Figure E in S1 File) adopts a planar conformation in the structure and packs against Trp309 (less than 3.5 Å away) as well as interacting with Lys65, Gln160, His219 and Asn304 (2.8–2.9 Å, ~3.2 Å, 2.6–2.7 Å and 2.5 to 3.0 Å, respectively; Fig 2B). Residue Trp309 packs flat against the malonate found in the structure presented here, whereas Trp309 is found at a significantly different orientation when there is no inhibitor in the active site as seen in 2QT3, suggesting that Trp309 moves in the occupied active site to provide interactions that improve substrate binding. The active sites of the independently solved structures show similar architectures, each having three histidine residues coordinated to the zinc ion (His60, His62, His217; 2.1 to 2.3 Å) as well as Asp303 (about 2.4 Å) and a water (2.0 to 2.1 Å) bound to the zinc (Fig 2B). The bound water is also within 2.7 Å of His249 and Asp303 as well as close to the bound malonate moiety. The 2QT3 structure has slightly longer bond lengths for all of the zinc interactions with the AtzC protein residues and water described above. The metal center is typical of a subtype III amidohydrolase (as defined by Seibert and Raushel [41,42]).

In the malonate:enzyme structure presented herein, additional electron density was found less than 3.5 Å from the malonate, above the plane and above the water molecule (about 4 Å) bound to the zinc ion. This density has been modelled as dimethylsulfoxide (DMSO), which was the solvent used for inhibitors and substrates which were used in both soaks and co-crystallization experiments. Oxygen atoms from both carboxylates of malonate are within hydrogen bonding distance of the water bound to zinc (2.4 to 2.8 Å) for both instances in the asymmetric unit.

The AtzC structure presented here is similar to that of the previously deposited AtzC structure (2QT3) with a 0.6 to 0.7 Å rmsd at the Cα level, respectively (Fig 3A and 3B). There is some divergence in the two structures between residues 37 to 43 and a more significant difference between the loop and helix seen at residues 79 to 97 (up to a 4.3 Å shift in Cα atoms in this region). These differences in structure impact upon the active site: residues Tyr82, Arg84 and Ile88 form part of the active site, which is less open in the structure presented here compared with 2QT3 due to the differences in the position of these residues. Tyr82 is part of a loop and Arg84 and Ile88 are a helix that sits above Trp309, which is tilted in 2QT3 relative to the Trp309 in our structure (which is almost flat-packed against the active site malonate).

The closest homologs to AtzC in the PDB (beyond 2QT3) are cytosine deaminases from *E*. *coli* and *K*. *pneumoniae*. *E*. *coli* cytosine deaminase (PDB 3R0D) has the greatest structure homology with the present structure and shows about 1.5 Å rmsd over the Cα backbone (17 gaps with 354 aligned residues using the SSM algorithm implemented in Coot [35]). *E*. *coli* cytosine deaminase (CodA) is a mononuclear Fe^2+^-dependent enzyme, albeit it has a high affinity for both zinc and iron [27,43,44]. The active sites of AtzC and CodA are similar with conservation of the three histidine and aspartate ligands, as well as several other residues (AtzC to 3R0D: His249 to His246, Gln160 to Gln156, Phe158 to Phe154, and Trp309 to Trp319, although this latter residue is in a different rotameric conformation in the two structures). However, there are several important residues that are different, including Asn304 (Asp314), His219 (Glu217), Lys65 (Thr66), Tyr82 (Leu81), Ser280 (Val278), Val310 (Tyr320) and there is a hole in the AtzC structure where Leu282 sits in the 3R0D structure, although this is partially occluded by Tyr82.

### Exploring the substrate-binding pocket

Although a malonate bound AtzC structure was obtained, the structure of AtzC with its substrate or product was elusive, despite concerted efforts. Although malonate is a weak inhibitor of AtzC (Figure E in S1 File), the high malonate concentration used in the crystallization trials likely prevented other small molecules from binding in the active site. A comparison of AtzC with 3O7U shows that the malonate overlays well with the CodA-bound (2R)-2-amino-2,5-dihydro-1,5,2-diazaphosphinin-6(1H)-one 2-oxide, an inhibitor of 3O7U: most of the atoms of the two inhibitors overlay within 0.5 A of each other after superposing the protein structures (Figure G in S1 File), with 6 of the atoms occupying similar positions (the oxygen in the ring of the inhibitor corresponding to one of the malonate carboxylate oxygen atoms and then 5 of the inhibitor ring atoms superposed with carbons and oxygens in the malonate). In fact the only atom of malonate that doesn’t correspond to an inhibitor atom is a carboxylate oxygen (that potentially hydrogen bonds to Lys65). As the CodA inhibitor binds CodA in an analogous manner to cytosine, the similarity in binding between 3O7U and its inhibitor and AtzC with malonate suggests that malonate may bind AtzC in a manner that is informative about the AtzC:*N*-isopropylammelide interaction.

**Fig 4:**
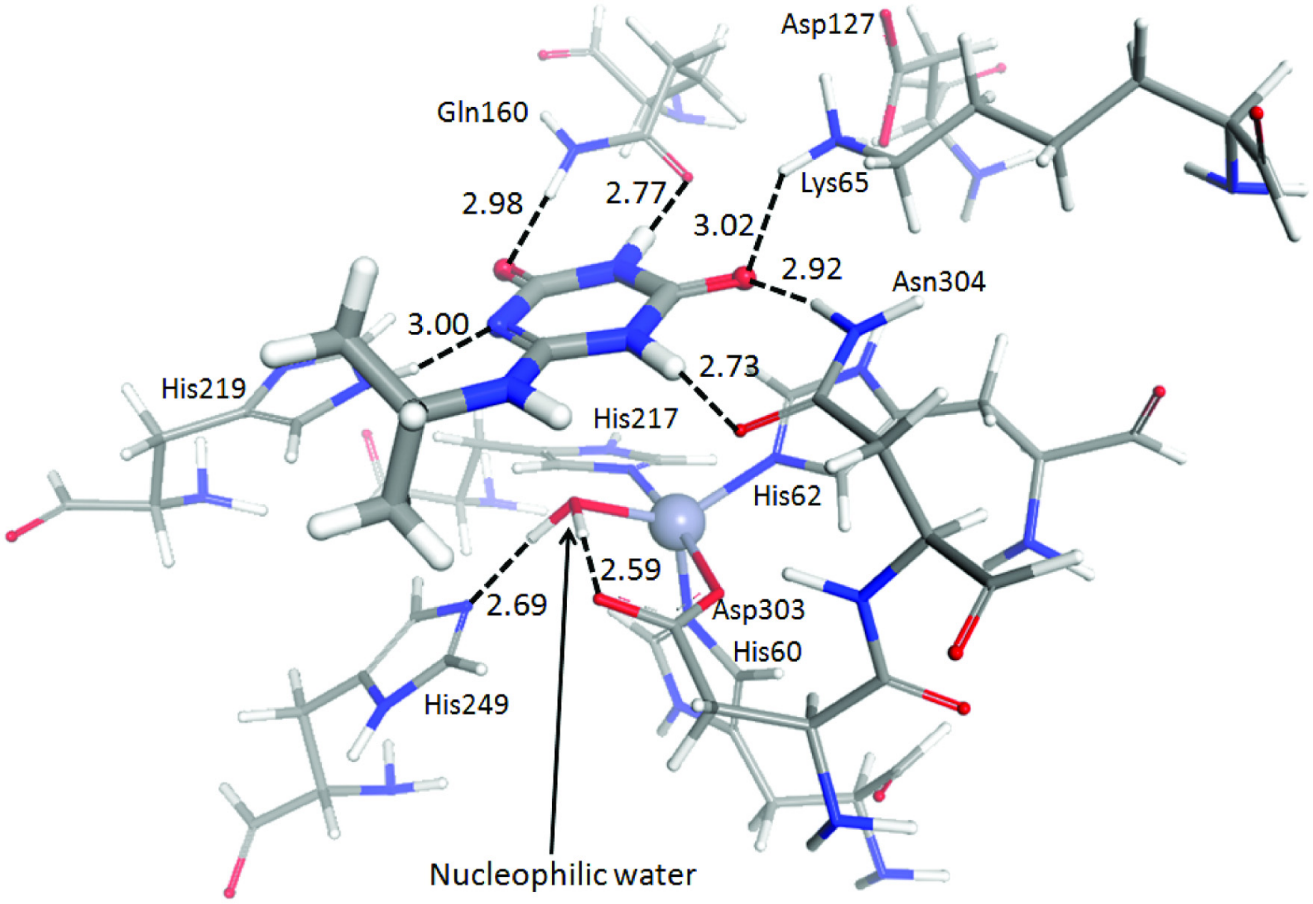
Docking the physiological substrate into the AtzC active site. Isopropylammelide (IPA, drawn with bonds of double thickness) docked into the active site of AtzC. The Zn^2+^ metal center is drawn as a grey sphere. Hydrogen bonding distances (between heteroatoms) are shown in Angstroms with the bonds themselves depicted as dashed lines.

Extensive attempts to find different crystallants more amenable to soaking or co-crystallization efforts were unsuccessful. Substrate docking, guided by the position of the inhibitor in the active site, revealed that five hydrogen-bonding interactions are likely to hold the substrate in a position facilitating nucleophilic attack at the isopropylamine-bearing carbon by the metal-bound nucleophile (Fig 4); Gln160 and Asn304 each donate and receive hydrogen bonds from IPA, Lys65 donates a hydrogen bond to the oxygen receiving a hydrogen bond from Asn304 and His219 donates a hydrogen bond to the aromatic nitrogen atom in the heterocycle. There is a tryptophan (Trp309, shown in Fig 2A) that sits directly above IPA in a π-stacking interaction that, during the geometry optimization, tilted approximately 1 Å out of its crystallographic position to accommodate the substrate. Trp309 has been shown to be motile in the comparison of the ligand-free AtzC structure and the inhibitor-bound structure, suggesting that this amino acid moves upon substrate binding. The N-H…O hydrogen bond donated by His219 is not perfectly linear and forces the hydrogen atom out of the plane of the ring of the side-chain, which would, in turn, exert an attractive force on the substrate in the direction of the nucleophile. The metal-bound water itself is donating hydrogen bonds to His249 and Asp303, the latter of which is also coordinated to the Zn^2+^ center. This orients the water with its lone pairs directed towards the substrate carbon bearing the isopropyl group.

The roles of substrate-binding pocket residues Lys65, Gln160, Asp188, His219, His249, Asp303, Asn304 and Trp309 were probed *via* mutagenesis of the *atzC* gene (Table 2). Crystal structures of WT, His219Ala, His249Ala and Asn304Asp have been deposited in the PDB (PDB: 4CQB, 4CQC, 4CQD and 5AKQ, respectively). Converting His249 to alanine resulted in a loss of detectable activity, although the DSF results confirmed that the protein was well-folded. This is suggests that His249 plays a pivotal role in the reaction mechanism of AtzC, perhaps in the generation of the metal-bound hydroxide as the nucleophile as an initial step. The pH optimum of AtzC is close to pH 7.25 (Figure C in S1 File), consistent with the suggested role of His249 in catalysis.

**Table 2:** Steady state kinetic characterization of AtzC and its variants. NDA: no detectable activity.

Variant	*k*_cat_ (s^-1^)	*K*_M_ (mM)	*K*_cat_/*K*_M_ (s^-1^.M^-1^)
**WT**	26.2 ± 1.8	3.1 ± 0.2	8.5 x 10^3^
**K65A**	778.5 ± 79.7	6.1 ± 0.6	1.3 x 10^5^
**K65R**	332.3 ± 34.3	2.4 ± 0.3	1.4 x 10^5^
**Q160A**	23.0 ± 3.5	1.8 ± 0.3	1.3 x 10^4^
**Q160E**	10.4 ± 0.5	1.3 ± 0.1	8.1 x 10^3^
**D188A**	106.5 ± 11.4	2.2 ± 0.2	4.9 x 10^4^
**D188N**	14.1 ± 3.1	1.9 ± 0.5	7.4 x 10^3^
**H219A**	16.8 ± 4	1.6 ± 0.4	1.1 x 10^4^
**H249A**	NDA	NDA	NDA
**D303A**	35.8 ± 3.5	1.7 ± 0.2	2.1 x 10^4^
**D303N**	90.4 ± 8.9	6.1 ± 0.6	1.5 x 10^4^
**N304A**	29.3 ± 1.7	4.2 ± 0.2	7.0 x 10^3^
**N304D**	189.0 ± 34	25.0 ± 4.5	7.6 x 10^3^
**W309A**	42.4 ± 4	5.6 ± 0.7	7.6 x 10^3^
**W309F**	21.3 ± 2	3.0 ± 0.3	7.1 x 10^3^

Substituting Lys65 to alanine gave a 30-fold increase in AtzC’s *k*_cat_ for ammelide. The Lys65Ala variant has one fewer hydrogen bonding interactions with the substrate than the wild-type, and it is possible that this may increase *k*_cat_ by increasing the *K*_D_ of AtzC for the product, thereby facilitating more rapid product egress. Similarly for the Lys65Arg substitution (12-fold increase in *k*_cat_ for ammelide), the product may be less-ideally bound due to the greater steric bulk of the arginine side chain, which would disrupt hydrogen bonding with the adjacent Asn304. Other mutations that reduced the number of hydrogen bonding interactions with the ring product are Asn304Asp (7-fold increase in *k*_cat_ for ammelide) and Asn304Ala (slight increase in *k*_cat_ for ammelide). Similarly, Trp309Ala opens up the area above the ring and increased the catalytic rate by almost a factor of two, which may further support the hypothesis that product departure is a rate limiting step for the hydrolysis of ammelide. Trp309Phe gave a very slight reduction in catalytic rate. Further work will be required to test the hypothesis that product departure is rate-limiting in AtzC.

Gln160 is involved in interactions with the substrate oxygen atom that, we propose, bears the negative charge during hydrolysis. When this residue was mutated to glutamate there was a reduction in *k*_cat_ of ~60%, which is likely due to the negative charge on the carboxylate impeding the development of the negative charge on the oxygen atom of the substrate which would otherwise facilitate catalysis. When the same residue was mutated to alanine, there was almost no change in *k*_cat_, which suggests that although it is positioned appropriately, the NH_2_ of the Gln160 side chain is not crucial for stabilizing the developing negative charge on the intermediate. Asp188 (not shown in Fig 4) lies within hydrogen bonding distance of His219 and its conversion to alanine increased the *k*_cat_ 4-fold, however conversion to asparagine reduced *k*_cat_ by nearly half. An asparagine in position 188 would most likely act as a hydrogen bond acceptor from both His219 and Arg84 (the latter not shown in Fig 4) given the proximity of one of the oxygen atoms on Asp188 to both residues. The inability of the asparagine oxygen atom to donate a hydrogen bond to His219 necessitates the latter to be doubly protonated in order to donate a hydrogen bond to the substrate as described above and exert the downwards force assisting nucleophilic attack by the coordinated water. This would not be the case if residue 188 was either alanine or aspartate, and this is reflected in the higher *k*_cat_ values observed for those cases. Furthermore, conversion of His219 to alanine reduced the *k*_cat_ by nearly the same amount as Asp188Asn, corroborating the geometries provided by the DFT calculations and our interpretation regarding the role of His219.

Asn304Asp provided a significant increase in *k*_cat_ (7-fold) but a corresponding increase in *K*_M_ to 25 mM (WT = 3.1 mM). This suggests that while this mutation provides a weaker product-binding site, it also significantly increases the proportion of non-productive substrate binding events. This is most likely due to the new unfavorable electrostatic interaction with the substrate oxygen. A transient repositioning of the nearby Lys65 to form an alternative salt bridge to the one between it and Asp127, sterically hindering substrate binding, is also possible. However, it should be noted that while the carbon atoms of the side chain of Lys65 are in a slightly different conformation in the crystal structure of the Asn304Asp mutant, the NH_3_^+^ group is occupying the same position.

### The AtzC reaction mechanism

**Fig 5:**
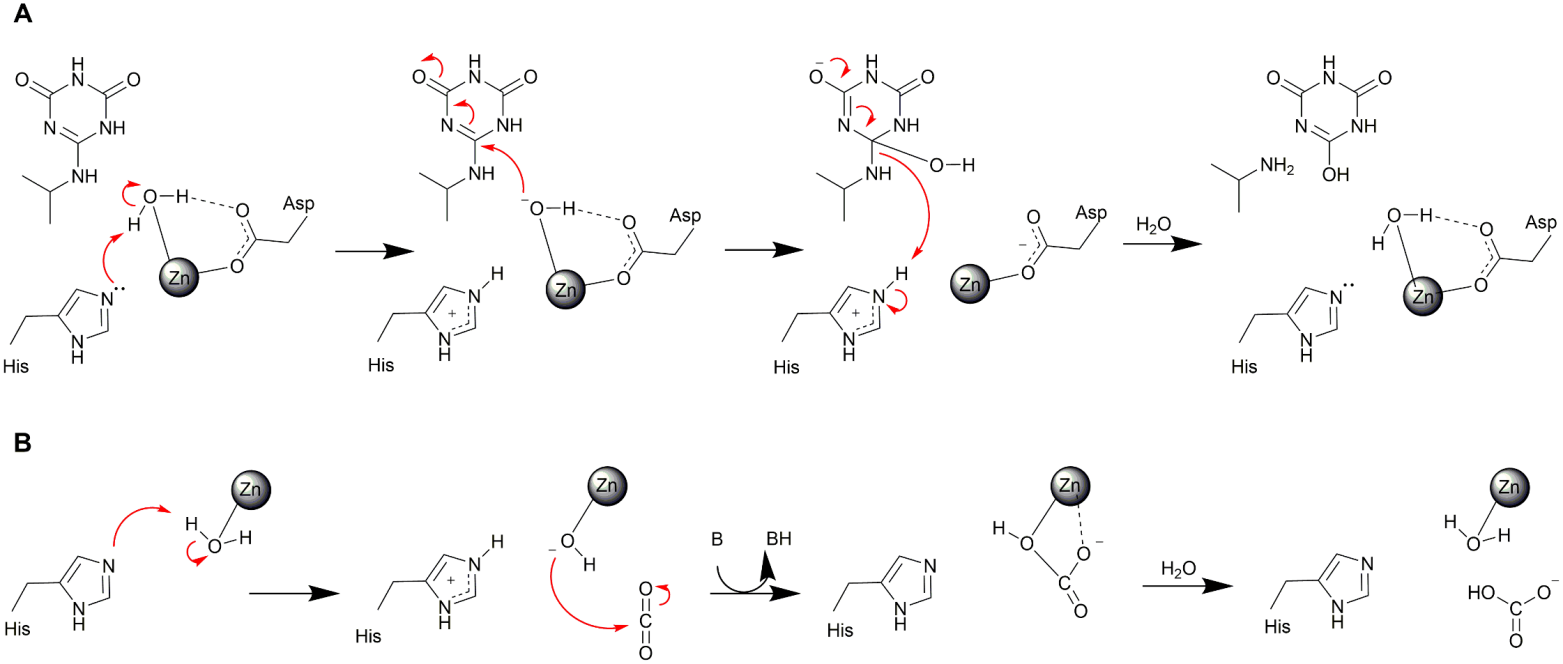
Reaction mechanism of AtzC and comparison with Carbonic anhydrase. The reaction mechanisms for: A) AtzC (proposed here) and B) carbonic anhydrase [45–47] are shown.

The mechanism proposed herein (Fig 5A), based on the X-ray crystal structure, docking, biochemical and mutagenesis studies with ammelide as substrate, appears straightforward. Conjugation on one side of the ring facilitates a shift of electron density onto the substrate oxygen atom receiving a hydrogen bond from the Gln160 side chain allowing nucleophilic attack to occur. A level of ambiguity arises in the first step, where the deprotonation of the metal-bound water could either occur concurrently with nucleophilic attack on the substrate, or occur in a two-step mechanism analogous to that of carbonic anhydrase [45–47] (Fig 5B), whereby in the latter, a stable, metal-bound hydroxide is created by His249, which acts as a proton shuttle, removing the proton and generating the active configuration (as shown in Fig 5A). Both mechanisms are plausible from the experimental and simulation data at hand, however the results of the mutagenesis favor the ‘carbonic anhydrase’ model. Notably this mechanism is fairly common (although far from universal) among the subtype III amidohydrolases [41], suggesting a predisposition for adopting the mechanism.

## Supporting Information

S1 File Figure A in S1 File. Crystals of AtzC grown in a PEG-based buffer (top) and a malonate-based buffer (bottom). The bar is approximately 200 μM in length. **Figure B in S1 File. Differential scanning fluorimetry of AtzC and its variants. Figure C in S1 File. pH dependency of AtzC-mediated ammelide deamination. Figure D in S1 File. Comparison of malonic acid and *N*-isopropylammelide.** Overlay of malonate (red) and IPA (blue) highlighting structural similarities between the two. There is excellent spatial overlap of three key H-bond acceptor groups (A) and an H-bond donor (D) which were key in guiding the choice of the starting pose prior for the DFT calculations. **Figure E in S1 File. Inhibition of AtzC by malonic acid. Figure F in S1 File. Docking malonic acid in AtzC active site**. The DFT optimised structure (cyan) with the crystal structure (purple). Backbone atoms were restrained and all other atoms were allowed to move freely, as in the original calculations. Hydrogen atoms are not shown for clarity. **Figure G in S1 File. Overlay of AtzC with bound malonate with CodA with bound inhibitor**. CodA (3O7U; green) with bound inhibitor ((2R)-2-amino-2,5-dihydro-1,5,2-diazaphosphinin-6(1H)-one 2-oxide; ADDO) was superposed with AtzC (cyan) using the SSM algorithm as implemented in Coot. 329 residues superpose with a rmsd of 1.52 Angstrom and 15 gaps (out of a possible 402/422 residues for 3O7U/AtzC). The sequence identity between the two proteins is 28.3%. Malonate (mal) in bound the AtzC active site and ADDO bound in the CodA active site are labelled. **Table A in S1 File. Oligonucleotides used in this study.**
(PDF)
