# Supplementary material for: X-Ray Structure and Mutagenesis Studies of the N-Isopropylammelide Isopropylaminohydrolase, AtzC
Source: PLoS One. 2015 Sep 21;10(9):e0137700. doi: 10.1371/journal.pone.0137700 (PMC4577212; doi:10.1371/journal.pone.0137700)
Supplement: S1 File — The bar is approximately 200 μM in length. Figure B in S1 File. Differential scanning fluorimetry of AtzC and its variants. Figure C in S1 File. pH dependency of AtzC-mediated ammelide deamination. Figure D in S1 File. Comparison of malonic acid and N-isopropylammelide. Overlay of malonate (red) and IPA (blue) highlighting structural similarities between the two. There is excellent spatial overlap of three key H-bond acceptor groups (A) and an H-bond donor (D) which were key in guiding the choice of the starting pose prior for the DFT calculations. Figure E in S1 File. Inhibition of AtzC by malonic acid. Figure F in S1 File. Docking malonic acid in AtzC active site. The DFT optimised structure (cyan) with the crystal structure (purple). Backbone atoms were restrained and all other atoms were allowed to move freely, as in the original calculations. Hydrogen atoms are not shown for clarity. Figure G in S1 File. Overlay of AtzC with bound malonate with CodA with bound inhibitor. CodA (3O7U; green) with bound inhibitor ((2R)-2-amino-2,5-dihydro-1,5,2-diazaphosphinin-6(1H)-one 2-oxide; ADDO) was superposed with AtzC (cyan) using the SSM algorithm as implemented in Coot. 329 residues superpose with a rmsd of 1.52 Angstrom and 15 gaps (out of a possible 402/422 residues for 3O7U/AtzC). The sequence identity between the two proteins is 28.3%. Malonate (mal) in bound the AtzC active site and ADDO bound in the CodA active site are labelled. Table A in S1 File. Oligonucleotides used in this study. (PDF) [file pone.0137700.s001.pdf]

**Fig. A.** Crystals of AtzC grown in a PEG-based buffer (top) and a malonate-based buffer (bottom). The bar is approximately 200 µM in length.

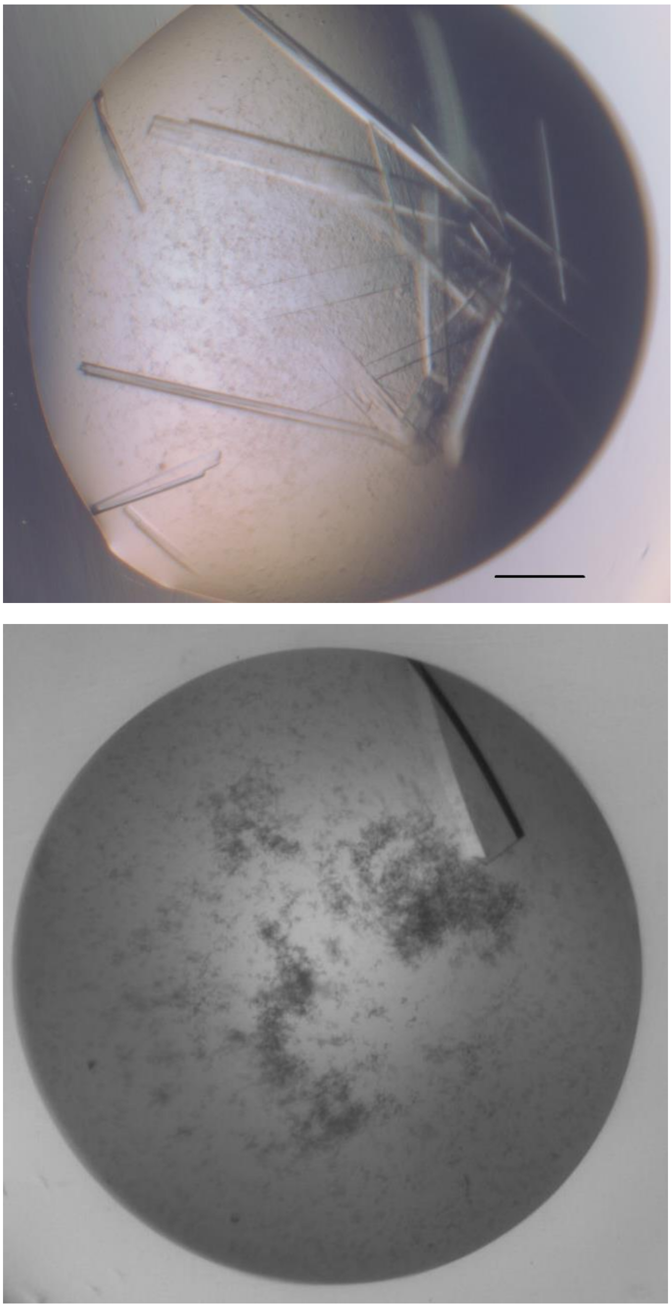

**Fig. B. Differential scanning fluorimetry of AtzC and its variants.**

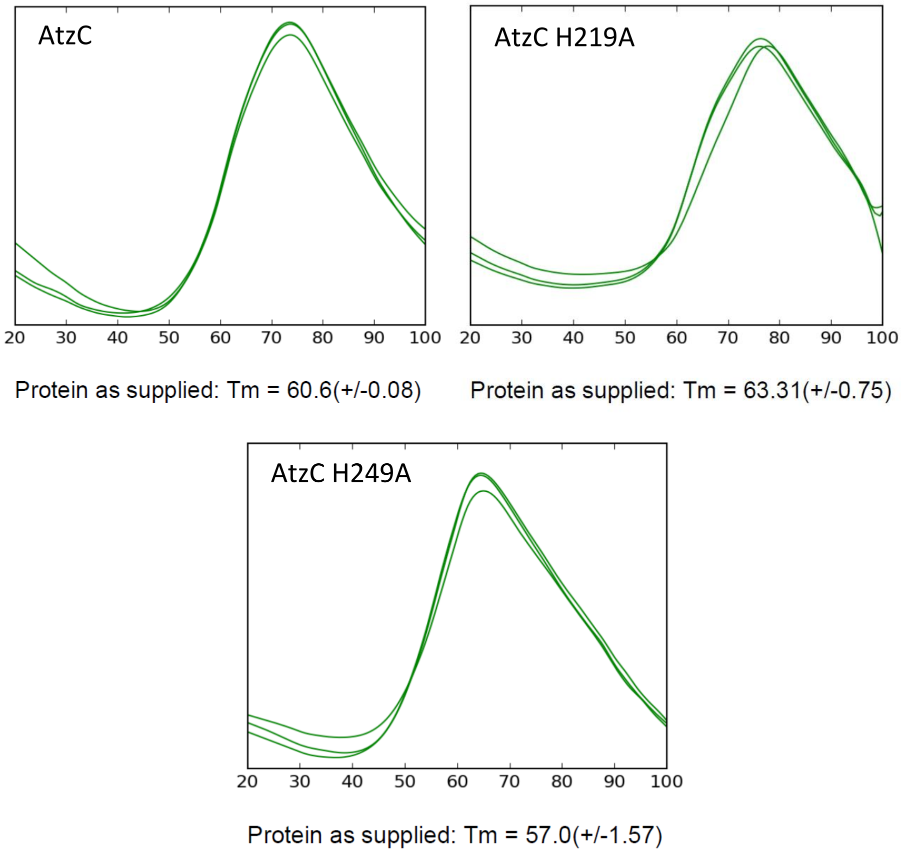

**Fig. C. pH dependency of AtzC-mediated ammelide deamination.**

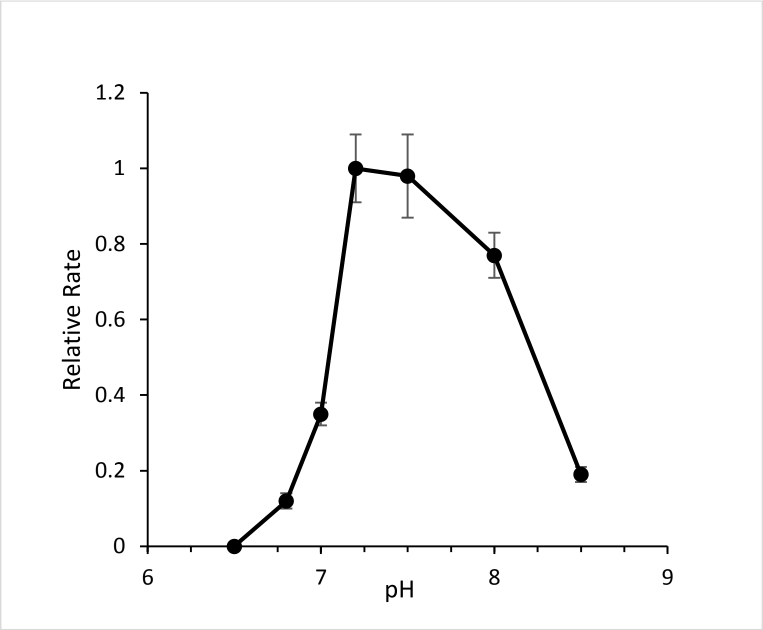

**Fig. D. Comparison of malonic acid and *N*-isopropylammelide**

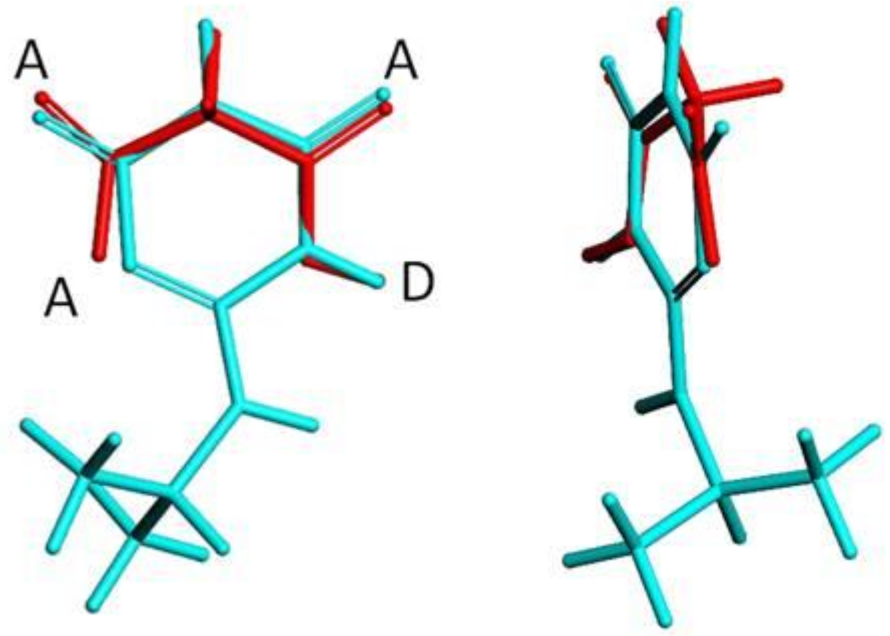


Overlay of malonate (red) and IPA (blue) highlighting structural similarities between the two. There is excellent spatial overlap of three key H-bond acceptor groups (A) and an H-bond donor (D) which were key in guiding the choice of the starting pose prior for the DFT calculations.

**Fig. E. Inhibition of AtzC by malonic acid.**

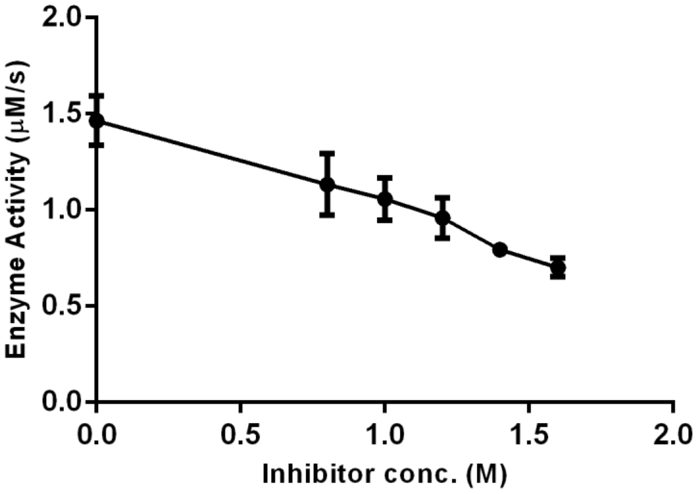

**Fig. F. Docking malonic acid in AtzC active site**. The DFT optimised structure (cyan) with the crystal structure (purple). Backbone atoms were restrained and all other atoms were allowed to move freely, as in the original calculations. Hydrogen atoms are not shown for clarity.

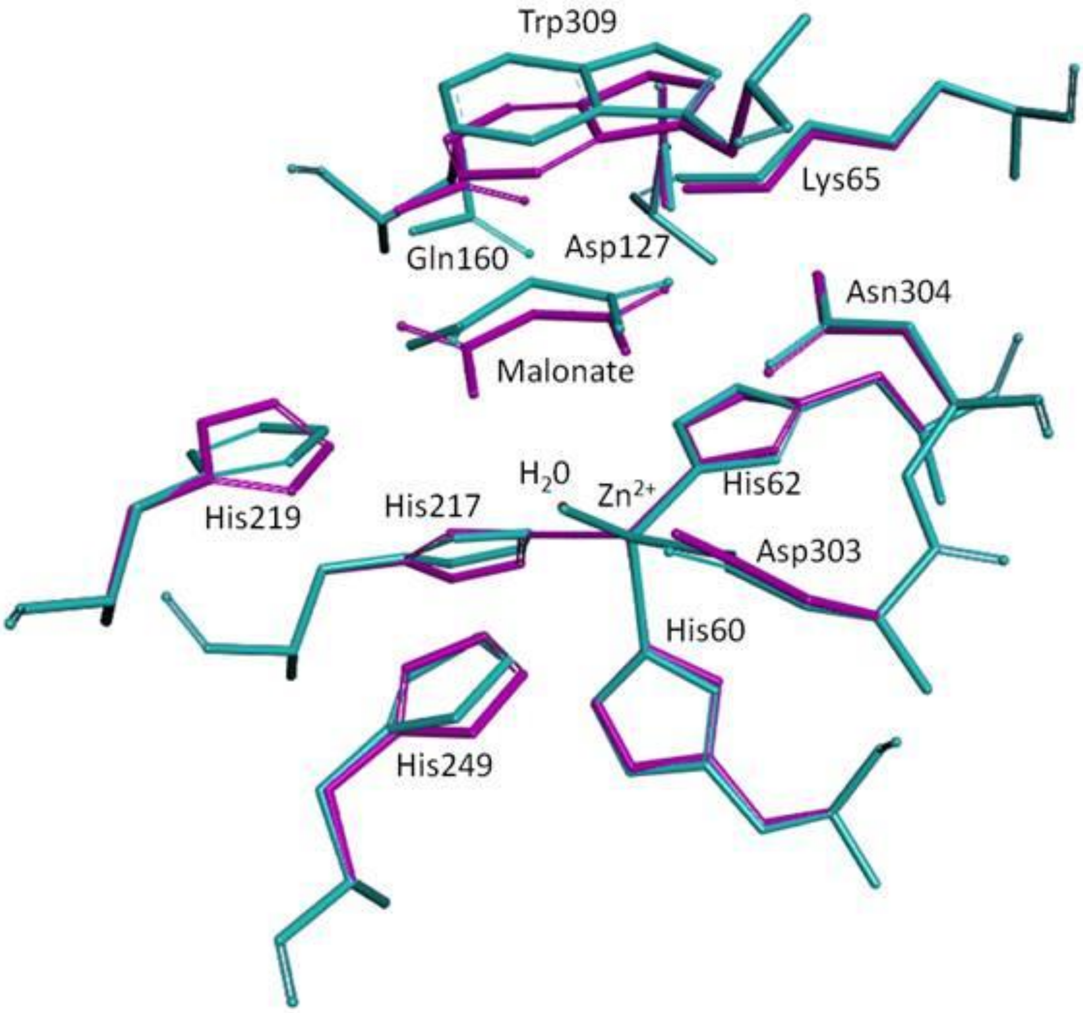

**Fig. G. Overlay of AtzC with bound malonate with CodA with bound inhibitor**. CodA (3O7U; green) with bound inhibitor ((2R)-2-amino-2,5-dihydro-1,5,2-diazaphosphinin-6(1H)-one 2-oxide; ADDO) was superposed with AtzC (cyan) using the SSM algorithm as implemented in Coot. 329 residues superpose with a rmsd of 1.52 Angstrom and 15 gaps (out of a possible 402/422 residues for 3O7U/AtzC). The sequence identity between the two proteins is 28.3%. Malonate(mal) in bound the AtzC active site and ADDO bound in the CodA active site are labelled.

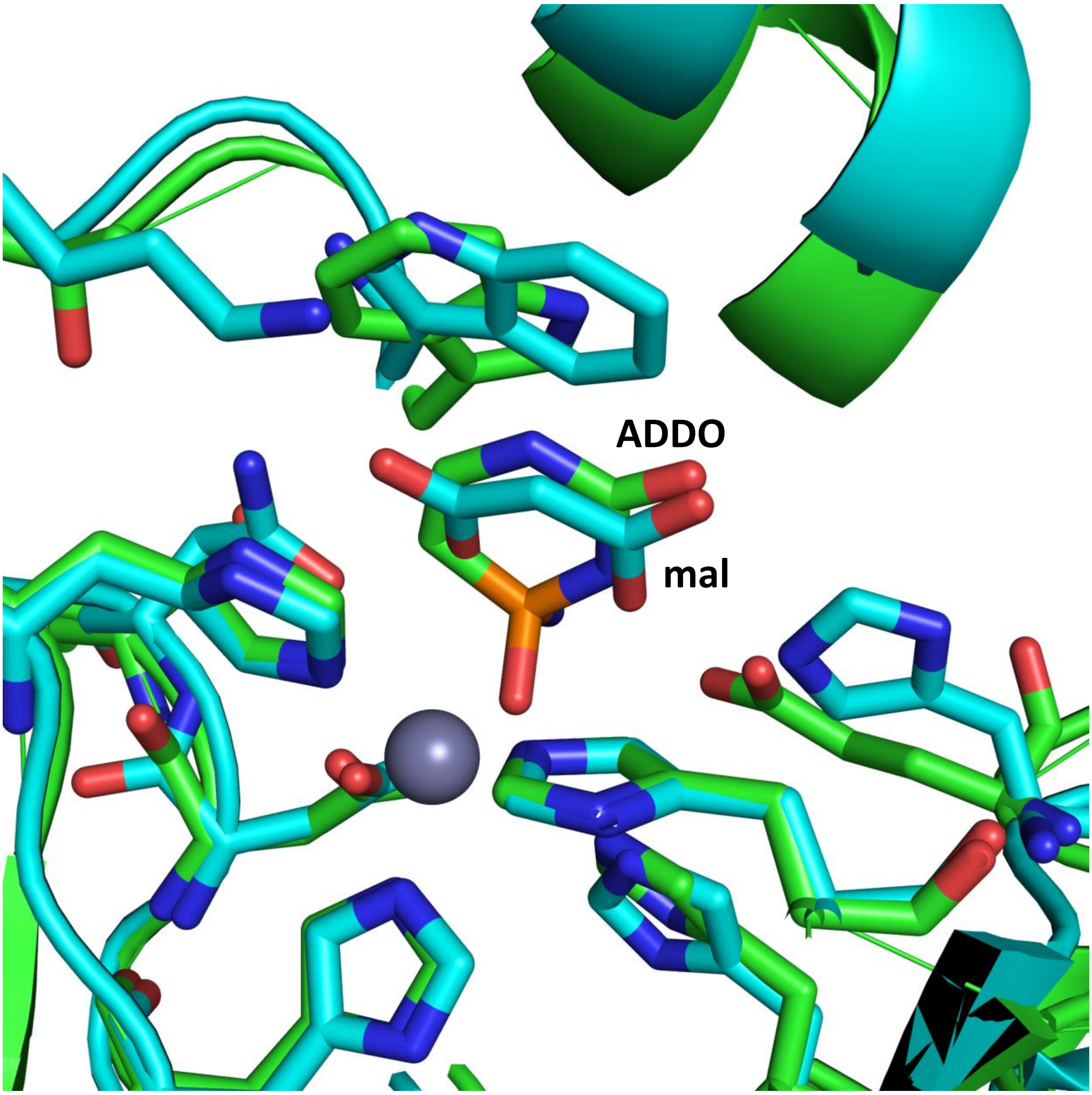

**Table A. Oligonucleotides used in this study.**

| Oligonucleotide name | Oligonucleotide sequence (5'-3') |
|---|---|
| **AtzC D303E fwd** | CTTGGCTGTGCTTCGGAAAATATCAGAGATTTTTGG |
| **AtzC D303E rev** | CAAAAATCTCTGATATTTTCCGAAGCACAGCCAAGA |
| **AtzC D303A fwd** | ATCTTGGCTGTGCTTCGGCGAATATCAGAGATTTTTG |
| **AtzC D303A rev** | CCAAAAATCTCTGATATTCGCCGAAGCACAGCCAAGA |
| **AtzC Q160N fwd** | GTCGTAGCCTTTGCAAATAGTGGATTTTTCGTTG |
| **AtzC Q160N rev** | AACGAAAAATCCACTATTTGCAAAGGCTACGACT |
| **AtzC Q160A fwd** | AGTCGTAGCCTTTGCAGCGAGTGGATTTTTCGTTG |
| **AtzC Q160A rev** | AACGAAAAATCCACTCGCTGCAAAGGCTACGACTT |
| **AtzC K65R fwd** | GCACATACCCACATGGATCGTTCATTTACGAGCACAGG |
| **AtzC K65R rev** | GTGTATGGGTGTACCTAGCAAGTAAATGCTCGTGTCCA |
| **AtzC K65A fwd** | ACATACCCACATGGATGCGTCATTTACGAGCACAG |
| **AtzC K65A rev** | TGTGCTCGTAAATGACGCATCCATGTGGGTATGTG |
| **AtzC N304Q fwd** | TTGGCTGTGCTTCGGACCAGATCAGAGATTTTTGGG |
| **AtzC N304Q rev** | ACCCAAAAATCTCTGATCTGGTCCGAAGCACAGCCA |
| **AtzC N304A fwd** | GGCTGTGCTTCGGACGCGATCAGAGATTTTTGGG |
| **AtzC N304A rev** | CCAAAAATCTCTGATCGCGTCCGAAGCACAGCCA |
| **AtzC D188E fwd** | GTTGGGGGAGTTGAACCTGCTACGCGGG |
| **AtzC D188E rev** | TCCCGCGTAGCAGGTTCAACTCCCCCAA |
| **AtzC D188A fwd** | TTGGGGGAGTTGCACCTGCTACGCG |
| **AtzC D188A rev** | CCGCGTAGCAGGTGCAACTCCCCCA |
| **AtzC W309F fwd** | GGACAATATCAGAGATTTTTTTGTTCCCTTTGGCAACGGTG |
| **AtzC W309F rev** | TCACCGTTGCCAAAGGGAACAAAAAAATCTCTGATATTGTC |
| **AtzC W309A fwd** | CAATATCAGAGATTTTGCGGTTCCCTTTGGCAACG |
| **AtzC W309A rev** | GTTGCCAAAGGGAACCGCAAAATCTCTGATATTGT |
| **AtzC H219A fwd** | TGATATCGACTATCACATAGCTGATATTGGAACTGTTGG |
| **AtzC H219A rev** | TCCAACAGTTCCAATATCAGCTATGTGATAGTCGATATC |
| **AtzC H249A fwd** | GGTAGAGTAACTACGAGTGCTGCCTGGTGTTTTGCAG |
| **AtzC H249A rev** | TCTGCAAAACACCAGGCAGCACTCGTAGTTACTCTAC |
